# Laparoscopy-assisted uterovaginal anastomosis in a patient with atypical cervicovaginal malformation: a case report

**DOI:** 10.3389/fmed.2025.1604463

**Published:** 2025-07-09

**Authors:** Antoine Naem, Graziella Moufawad, Suzana Sultan, Zaki Sleiman

**Affiliations:** ^1^Faculty of Mathematics and Computer Science, University of Bremen, Bremen, Germany; ^2^Lebanese American University Medical Center –Rizk Hospital, Beirut, Lebanon; ^3^Faculty of Medicine of Tishreen University, Latakia, Syria

**Keywords:** anastomosis, cervicovaginal agenesis, fertility preservation, laparoscopy, Müllerian anomalies, reconstructive surgery, uterine-sparing

## Abstract

Cervicovaginal anomalies are rare and form 4–7% of the Müllerian anomalies. Traditionally, cervicovaginal agenesis/dysgenesis had been treated by hysterectomy due to the high risks of restenosis and sepsis which are associated with cervical canalization. In this work, we report the case of a 14-year-old patient who presented with amenorrhea and cyclic abdominal pain. The patient had normal secondary sexual development. Magnetic resonance imaging revealed hematometra and bilateral hematosalpinx. Laparoscopic exploration identified an obstructed cervix and blunt vagina. The patient underwent direct cervicovaginal anastomosis under laparoscopic guidance. After 1 month of follow-up, the patient had normal menstruation and a healthy uterine cavity and cervical canal upon hysteroscopy.

## 1 Introduction

The Müllerian anomalies are relatively rare with a prevalence of 1 per 4,000–10,000 women (1). Cervicovaginal anomalies, on the other hand, are an even rarer subtype of the Müllerian malformations with a reported prevalence between 4–7% (2). The cervicovaginal anomalies result from a defective lengthening or vacuolization of the Müllerian ducts (3, 4). On this basis, Cervicovaginal anomalies are broadly classified into cervical agenesis and cervical dysgenesis (5). Cervical agenesis refers to the complete absence of the uterine cervix and cervical canal, whereas cervical dysgenesis refers to the presence of an abnormal cervix or cervical canal. Cervical dysgenesis is further subclassified into 4 subtypes, these are namely: cervical obstruction, a cervical body consisting of a fibrous band, cervical fragmentation, and stricture of the mid-portion of the uterine cervix (6). It is noteworthy that vaginal aplasia coexists with cervicovaginal anomalies in nearly 39% of cases (7).

Affected patients typically present with primary amenorrhea and cyclic abdominal pain (4). Such cases were classically treated by hysterectomy due to the high complication rate of cervical drilling and the associated morbidity and mortality risks (8). However, a general tendency to adopt conservative management strategies has been observed in recent years due to the increased experience with and improved technologies of laparoscopic surgery (9, 10). Uterovaginal and utero-vestibular anastomosis are conservative reconstructive procedures that aim to restore the continuity of the reproductive tract via direct suturing (4). In this case, we demonstrate the surgical management of an adolescent with cervicovaginal dysgenesis along with her follow-up results.

## 2 Case description

**Figure 1:**
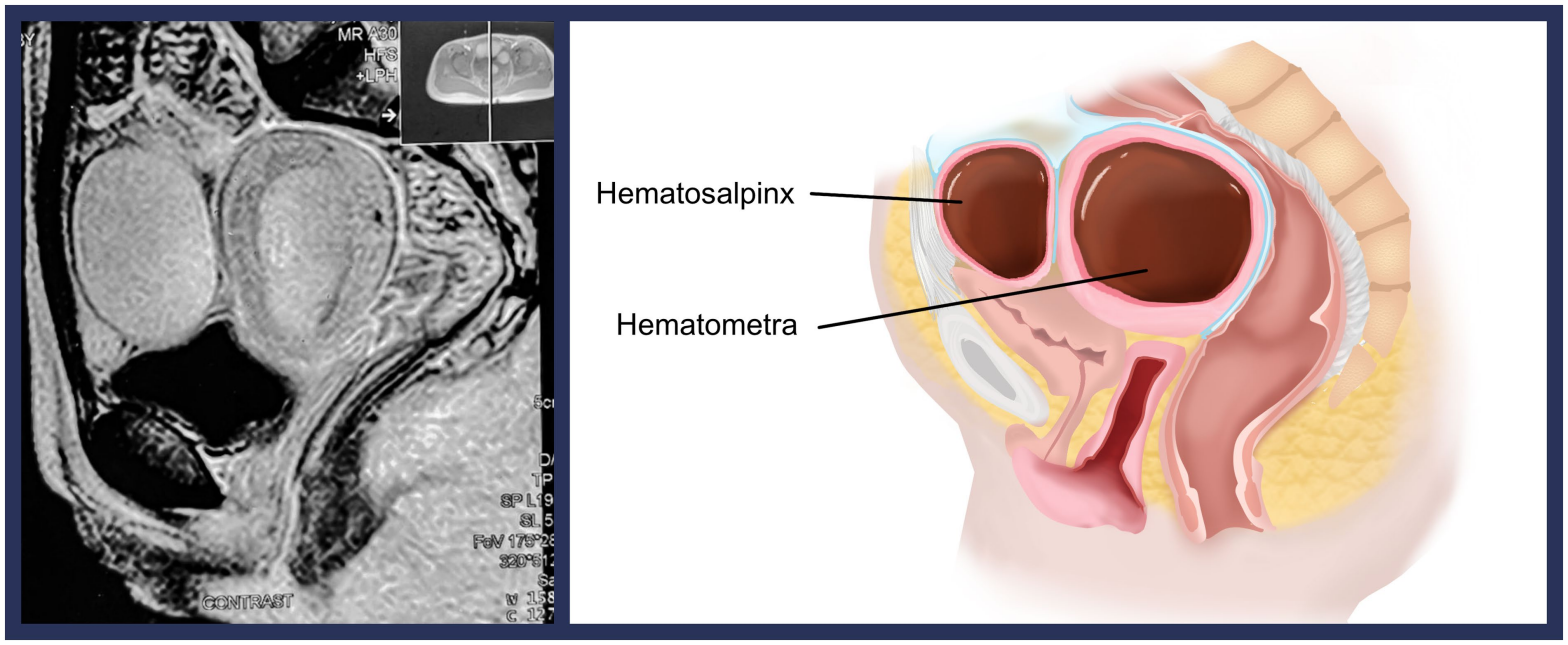
Magnetic resonance imaging demonstrating the hypertrophic uterus with the hematometra and the left hematosalpinx (left). Additionally, a visual demonstration of the malformation is presented (right).

A 14-year-old adolescent girl presented with primary amenorrhea and cyclic abdominal pain. The gynecologic examination revealed normally developed external genitalia and a partially aplastic vagina measuring 4 cm in length and 2 cm in width. Magnetic resonance imaging (MRI) revealed an enlarged uterus with hematometra and bilateral hematosalpinx (Figure 1). The radiologists were unable to decide on the presence or absence of the cervical tissue based on the MRI images. The MRI did not show any urologic abnormality. After careful discussion with the patient and her parents, the patient was scheduled to undergo laparoscopic surgery to better assess her condition and treat the underlying obstructive anomalies.

The patient was placed in the dorsal lithotomy position. Under general anesthesia and after achieving proper intra-abdominal pressure, standard trocar placement took place. A 12-mm umbilical trocar for the laparoscope and two working 10-mm trocars at the level of the anterior superior iliac spines were placed via the direct entry method.

Initial exploration of the pelvic cavity revealed bilateral hematosalpinx. Moreover, the cervical tissue could be observed but it was discontinued with the vagina. Endometriosis lesions and intrapelvic adhesions were absent.

**Figure 2:**
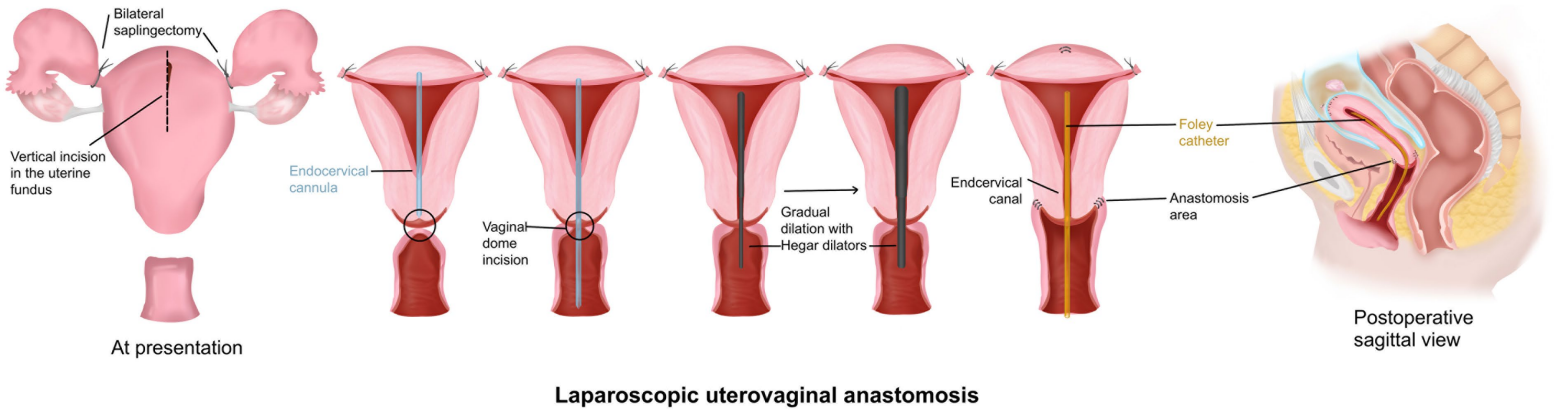
Laparoscopy-assisted uterovaginal anastomosis. A summary of the key surgical steps is illustrated.

**Figure 3:**
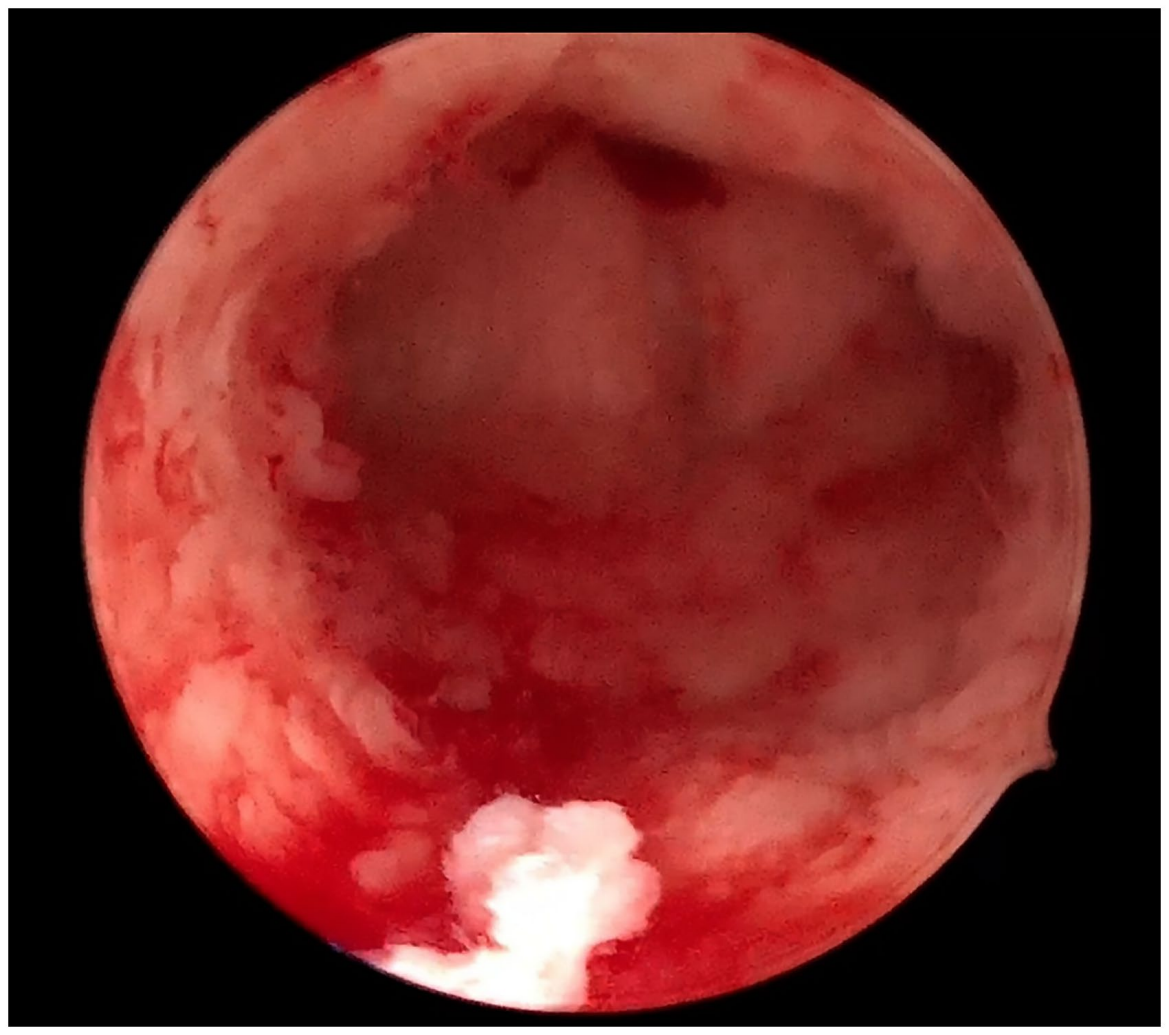
The hysteroscopic appearance of the endocervical canal after 4 weeks of follow-up.

Firstly, bilateral salpingectomies were performed laparoscopically. To minimize bleeding, bilateral clipping of the uterine arteries was done. Afterward, the uterine fundus was incised vertically with a monopolar hook. The hematometra was drained using the suction cannula. Under laparoscopic guidance, the cannula was driven through the endocervical canal toward the vaginal dome. This step aims to better identify the uterovaginal axis and canalize the obstructed endocervical canal. The cannula was palpated transvaginally at the top of the vaginal dome, where a small horizontal incision was made. The uterine cervix was dilated gradually with Hegar dilators until a dilator size of 8 was reached. The uterine cervix was then sutured to the vagina with 2–0 Vicryl stitches. Thereafter, a 17F Foley catheter was introduced to the uterine cavity in the railroad fashion. Eventually, the hysterotomy incision was closed and the uterine arteries were unclipped. The surgical procedure is summarized in Figure 2 and detailed with a video footage in Supplementary Video 1. No intra-or postoperative complications occurred. The intrauterine catheter was kept for 2 weeks to prevent cervical stenosis. After 4 weeks of the initial operation, an exploratory hysteroscopy revealed a healthy cervical canal and uterine cavity. In addition, the patient had normal menstruation by the time of follow-up, and she was symptom-free (Figure 3).

## 3 Discussion

We presented a case of cervical atresia and partial vaginal aplasia in a 14-year-old patient. The patient was managed laparoscopically with a uterine-sparing reconstructive technique. At follow-up, the patient was symptom-free with an anatomically normal uterine cavity. In our opinion, successful management of cervicovaginal anomalies should relieve the patient’s symptoms and restore the continuity between the uterine cavity, cervical canal, and vagina in order to preserve the patient’s fertility. Nevertheless, an early diagnosis is crucial to achieve a successful surgical outcome and to avoid the psychological burden of this entity on the patient. A recent study by Liu et al. (11) demonstrated that half of the patients with cervicovaginal malformations suffer from depressive symptoms and 58.7% of them exhibit anxiety symptoms. This study also revealed that patients with depression were older and had longer preoperative pain periods compared to non-depressed patients, which encourages an early diagnosis and management (11).

A functional uterine cavity is present in 2–7% of patients with Müllerian anomalies (1). Considering that orthograde menstruation begins at menarche, delayed diagnosis and treatment theoretically increase the risk of endometriosis, intrapelvic adhesions, and inflammation (4). The key to an early diagnosis is keeping a high level of suspicion of cervicovaginal agenesis/dysgenesis and discriminating it from other pathologies that share the same presentation, such as imperforated hymen and obstructive transvaginal septum. Most patients with cervicovaginal anomalies present with primary amenorrhea and cyclic abdominal pain (2). In such cases, transabdominal -or when applicable, transvaginal- ultrasonography and MRI are valuable to determine the level of obstruction. An MRI is also useful to detect most coexisting urologic anomalies since it was reported in 15–20% of patients with cervicovaginal malformations (3).

Due to the complexity of the surgical reconstruction of these malformations, a direct intervention after the diagnosis may not be feasible. Therefore, some authors recommend hormonal suppression with oral contraceptives or Gonadotropin-releasing Hormone (GnRH) agonists to stop orthograde menstruation and associated etiologic processes (4).

A standardized surgical intervention for cervicovaginal anomalies is hard to determine owing to the vast heterogeneity of these malformations. Fedele et al. (4) indicated that the type of surgical intervention should be determined based on the cervical anomaly type and the presence or absence of the vagina. In fact, an earlier systematic review demonstrated that regardless of the surgical approach, the presence of a congenitally healthy vagina improved the postoperative pregnancy chances (2). Furthermore, Mikos et al. (2) suggested that a practical and comprehensive classification of uterovaginal anomalies should take into account the presence or absence of a normal vagina, which is unfortunately not explicitly implemented in some current classification systems of the Müllerian anomalies. The joint classification of Müllerian anomalies by the European Society of Human Reproduction and Embryology and the European Society of Gynecologic Endoscopy (ESHRE/ESGE) (12) covers the possible vaginal malformations but unfortunately misses many cervical malformations that were mentioned by Rock et al. (6). In our opinion, a revised ESHRE/ESGE classification of the Müllerian anomalies that covers all potential uterocervical malformations would be of great clinical value.

Many authors had recommended hysterectomy to be the treatment of choice for patients with cervicovaginal anomalies due to the elevated risk of restenosis and sepsis (8). Interestingly, the first attempt to conservatively repair a cervicovaginal anomaly dates to 1900 (13). Recent studies demonstrated the safety and effectiveness of surgical reconstructions for patients with Müllerian anomalies. The conservative surgical management of uterovaginal agenesis/dysgenesis could be broadly classified into 3 groups: cervical canalization, uterovaginal or utero-vestibular anastomosis, and transplantation of homologous or heterologous grafts (4). In the systematic review of Mikos et al. (2), the anastomotic procedures were the most commonly implemented. Most of the patients who underwent uterovaginal anastomosis had either cervical agenesis or cervical obstruction. The postoperative menstruation rate was 93.3%. Additionally, the authors reported a postoperative pregnancy rate of 14.1% (2). These results elucidate the effectiveness of this procedure for the treatment of cervical malformations.

Direct comparisons between these techniques are not available due to the scarcity of cervicovaginal agenesis/dysgenesis cases. However, it is our belief that comparisons have little to no value at all, due to the heterogenous spectrum of cervicovaginal anomalies and associated malformations. The surgical intervention should be as personalized as possible and tailored to the patient’s initial presentation.

## 4 Conclusion

The cervicovaginal anomalies are a rare clinical entity that has severe consequences on the patient’s mental and physical health. Laparoscopic cervicovaginal anastomosis is a feasible technique that restores the continuity between the cervix and the vagina and preserves the patient’s fertility with an acceptable safety profile. Conservative reconstructive surgeries should be considered as a first treatment in patients with cervicovaginal agenesis or dysgenesis.

## Data Availability

The original contributions presented in the study are included in the article/Supplementary material, further inquiries can be directed to the corresponding author.
